# Relative Termination via Dependency Pairs

**DOI:** 10.1007/s10817-016-9373-5

**Published:** 2016-04-30

**Authors:** José Iborra, Naoki Nishida, Germán Vidal, Akihisa Yamada

**Affiliations:** 1 London, UK; 2 Graduate School of Information Science, Nagoya University, Nagoya, Japan; 3 MiST, DSIC, Universitat Politècnica de València, Valencia, Spain; 4 Institute of Computer Science, University of Innsbruck, Innsbruck, Austria

**Keywords:** Term rewriting, Dependency pairs, Termination

## Abstract

A term rewrite system is terminating when no infinite reduction sequences are possible. Relative termination generalizes termination by permitting infinite reductions as long as some distinguished rules are not applied infinitely many times. Relative termination is thus a fundamental notion that has been used in a number of different contexts, like analyzing the confluence of rewrite systems or the termination of narrowing. In this work, we introduce a novel technique to prove relative termination by reducing it to dependency pair problems. To the best of our knowledge, this is the first significant contribution to Problem #106 of the RTA List of Open Problems. We first present a general approach that is then instantiated to provide a concrete technique for proving relative termination. The practical significance of our method is illustrated by means of an experimental evaluation.

## Introduction

Analyzing whether a program terminates or not is a fundamental problem that has been extensively studied in almost all programming paradigms. For *term rewrite systems (TRSs)*, termination analysis has attracted considerable attention (see, e.g., the survey by Zantema [37] and the termination portal^1^), and various automated termination provers for TRSs have been developed: AProVE  [11],  [24], NaTT  [34], etc. Among them the *dependency pair (DP) method* [2, 13] and its successor the *DP framework* [12] became a modern standard.

Termination of a TRS is usually checked for all possible reduction sequences. In some cases, however, one is interested in a generalized notion, called *relative termination* [9, 21]. Roughly speaking, a TRS $\mathcal{R}$ is relatively terminating w.r.t. another TRS $\mathcal{B}$, which we call the *base* in this paper, when any infinite reduction using both systems contains only a finite number of steps given with rules from $\mathcal{R}$. For instance, consider the following base:

$$\begin{aligned} \mathcal{B}_\mathsf {comlist} = \{\; \mathsf {cons}(x,\mathsf {cons}(y,ys)) \rightarrow \mathsf {cons}(y,\mathsf {cons}(x,ys))\; \} \end{aligned}$$

which specifies the property of commutative lists (i.e., that the order of elements is irrelevant). Termination of operations on commutative lists, described by a TRS $\mathcal{R}$, can be analyzed as the relative termination of $\mathcal{R}$ w.r.t. $\mathcal{B}_\mathsf {comlist}$. Note also that the base $\mathcal{B}_\mathsf {comlist}$ is clearly non-terminating.

In this paper, we present a new technique for proving relative termination by reducing it to the finiteness of dependency pair problems. To the best of our knowledge, we provide the first significant contribution to Problem #106 of the *RTA List of Open Problems* 2:

Can we use the dependency pair method to prove relative termination?

Relative termination has already been used in various contexts: proving confluence of a rewrite system [9, 16]; liveness properties in the presence of fairness [23]; and termination of narrowing [18, 29, 33], an extension of rewriting to deal with non-ground terms (see, e.g., [17]). Moreover, analyzing relative termination can also be useful for other purposes, like dealing with random values or considering rewrite systems with so-called *extra-variables* (i.e., variables that occur in the right-hand side of a rule but not in the corresponding left-hand side). For instance, the following base $\mathcal{B}_\mathsf {rand}$ specifies a random number generator:

$$\begin{aligned} \mathcal{B}_\mathsf {rand} = \{\,\mathsf {rand}(x) \rightarrow x,\ \mathsf {rand}(x) \rightarrow \mathsf {rand}(\mathsf {s}(x))\,\} \end{aligned}$$

We have $\mathsf {rand}(\mathsf {0}) \rightarrow _{\mathcal{B}_\mathsf {rand}}^{*} \mathsf {s}^n(\mathsf {0})$ for arbitrary $n \in {\mathbb {N}}$. Now, consider

$$\begin{aligned} \begin{array}{lllrcl@{\quad }rcll} \mathcal{R}_\mathsf {quot} &{} = &{} \{ &{} x - \mathsf {0} &{}\rightarrow &{} x, &{}\mathsf {s}(x) - \mathsf {s}(y) &{}\rightarrow &{} x - y,\\ &{}&{}&{}\mathsf {quot}(\mathsf {0},\mathsf {s}(y)) &{}\rightarrow &{} \mathsf {0}, &{}\mathsf {quot}(\mathsf {s}(x),\mathsf {s}(y)) &{}\rightarrow &{} \mathsf {s}(\mathsf {quot}(x-y,\mathsf {s}(y))) &{} \}\\ \end{array} \end{aligned}$$

from Arts and Giesl [2]. Termination of $\mathcal{R}_\mathsf {quot}$ can be shown using the DP method [2]. However, using previously known techniques, it is not possible to prove that $\mathcal{R}_\mathsf {quot}$ is relatively terminating w.r.t. $\mathcal{B}_\mathsf {rand}$. Note also that the relative termination of $\mathcal{R}_\mathsf {quot}$ w.r.t. $\mathcal{B}_\mathsf {rand}$ is not at all obvious, since $\mathcal{R}_\mathsf {quot}$ is not relatively terminating w.r.t. the following similar variant $\mathcal{B}_\mathsf {gen}$:

$$\begin{aligned} \mathcal{B}_\mathsf {gen} = \{\,\mathsf {gen} \rightarrow \mathsf {0},\ \mathsf {gen} \rightarrow \mathsf {s}(\mathsf {gen})\,\} \end{aligned}$$

which is considered in the context of termination of narrowing [18, 29, 33]. Indeed, we can construct the following infinite reduction sequence using $\mathcal{B}_\mathsf {gen}$:

$$\begin{aligned} \mathsf {s}(\mathsf {gen}) - \mathsf {s}(\mathsf {gen}) \rightarrow _{\mathcal{R}_\mathsf {quot}} \mathsf {gen} - \mathsf {gen} \rightarrow _{\mathcal{B}_\mathsf {gen}}^{*} \mathsf {s}(\mathsf {gen}) - \mathsf {s}(\mathsf {gen}) \rightarrow _{\mathcal{R}_\mathsf {quot}} \cdots \end{aligned}$$

We expect our technique to be also useful to deal with TRSs with extra-variables. In principle, these systems are always non-terminating, since extra-variables can be replaced by any term. However, one can still consider an interesting termination property: Is the system terminating if the extra-variables can only be instantiated with terms built from a restricted signature? Consider, e.g., the following TRS from [28]:

$$\begin{aligned} \mathcal{R}= \{ ~{\mathsf {f}}(x, {\mathsf {0}}) \rightarrow {\mathsf {s}}(x),\ {\mathsf {g}}(x) \rightarrow {\mathsf {h}}(x, y),\ {\mathsf {h}}({\mathsf {0}}, x) \rightarrow {\mathsf {f}}(x, x), \ {\mathsf {a}} \rightarrow \mathsf {b} ~\} \end{aligned}$$

This system is clearly non-terminating due to the extra variable in the second rewrite rule. However, by assuming that *y* can only take values built from constructor symbols (e.g., natural numbers), one can reformulate these rewrite rules as follows:

$$\begin{aligned} \mathcal{R}' = \{ ~{\mathsf {f}}(x, {\mathsf {0}}) \rightarrow {\mathsf {s}}(x),\ {\mathsf {g}}(x) \rightarrow {\mathsf {h}}(x, \mathsf {gen}),\ {\mathsf {h}}({\mathsf {0}}, x) \rightarrow {\mathsf {f}}(x, x),\ {\mathsf {a}} \rightarrow \mathsf {b} ~\} \end{aligned}$$

using $\mathcal{B}_{\mathsf {gen}}$ above. Obviously, $\mathcal{R}'\cup \mathcal{B}_{\mathsf {gen}}$ is still non-terminating, since $\mathcal{B}_{\mathsf {gen}}$ is already non-terminating. Nevertheless, one can still prove the relative termination of $\mathcal{R}'$ w.r.t. $\mathcal{B}_{\mathsf {gen}}$, which is an interesting property since one can ensure terminating derivations by using an appropriate heuristics to instantiate extra-variables.

Another interesting application of relative termination is *test case generation*. For example, in the QuickCheck tool for property-based testing, lists over, e.g., natural numbers are generated randomly. Assume *f* and *g* are defined externally by a TRS $\mathcal{R}_{ fg }$, and consider the TRS $\mathcal{R}_ test$ consisting of the following rules:

$$\begin{aligned} {\mathsf {rands}}({\mathsf {0}},y)&\rightarrow \mathsf {done}(y)&{\mathsf {eq}}(x,x)&\rightarrow \mathsf {true} \\ {\mathsf {rands}}({\mathsf {s}}(x),y)&\rightarrow {\mathsf {rands}}(x, \mathsf {cons}(\mathsf {rand}({\mathsf {0}}), y))\\ {\mathsf {tests}}({\mathsf {0}})&\rightarrow {\mathsf {true}}&{\mathsf {true}} \wedge y&\rightarrow y \\ {\mathsf {tests}}({\mathsf {s}}(x))&\rightarrow {\mathsf {test}}(\mathsf {rands}(\mathsf {rand}({\mathsf {0}}),{\mathsf {nil}})) \wedge \mathsf {tests}(x)&{\mathsf {false}} \wedge y&\rightarrow \mathsf {false} \\ {\mathsf {test}}(\mathsf {done}(y))&\rightarrow \mathsf {eq}(f(y),g(y)) \end{aligned}$$

where lists are built from $\mathsf {nil}$ and $\mathsf {cons}$. Execution of $\mathsf {tests}({\mathsf {s}}^n({\mathsf {0}}))$ tests the equivalence between *f* and *g* by feeding them random inputs *n* times. Even when *f* and *g* are defined by $f(x) \rightarrow x$ and $g(x) \rightarrow x$, AProVE fails to prove the relative termination of $\mathcal{R}_ test \cup \mathcal{R}_{ fg }$ w.r.t. $\mathcal{B}_\mathsf {rand}$.

In this paper, we propose a method to prove relative termination using the dependency pair framework. We implemented the proposed method in the termination tool NaTT 3 and show its significance through experiments. Using results of this paper and [35], NaTT can prove the relative termination of $\mathcal{R}_\mathsf {quot}$ w.r.t. $\mathcal{B}_\mathsf {rand}$, and that of $\mathcal{R}_ test \cup \mathcal{R}_{ fg }$ w.r.t. $\mathcal{B}_\mathsf {rand}$ for, e.g., naive and tail recursive definitions of summation as *f* and *g*, respectively.

This paper is organized as follows. In Sect. 2, we briefly review some notions and notations of term rewriting. In Sects. 3–5, we present our main contributions for reducing relative termination to a dependency pair problem. In Sect. 6, we show that the subterm criterion and the usable rule argument is applicable under certain conditions. Then, Sect. 7 describes implementation issues , and presents selected results from an experimental evaluation. Finally, Sect. 8 compares our technique with some related work, and Sect. 9 concludes and points out some directions for future research.

## Preliminaries

We assume some familiarity with basic concepts and notations of term rewriting. We refer the readers to, e.g., [4] for further details. Here we recall some fundamental notions needed in this paper.

A *signature* $\mathcal{F}$ is a set of function symbols. Given a set of *variables* $\mathcal{V}$ with $\mathcal{F}\cap \mathcal{V}=\emptyset$, we denote the domain of *terms* by $\mathcal{T}(\mathcal{F},\mathcal{V})$. We use ${\mathsf {f}},{\mathsf {g}},\ldots$ to denote function symbols and $x,y,\ldots$ to denote variables. The *root symbol* of a term $t = f(t_1,\dots ,t_n)$ is *f* and denoted by $\mathsf {root}(t)$. We assume an extra fresh constant $\Box$ called a *hole*. A *context* is a term $C[\,]$ where the hole occurs exactly once. We write *C*[*t*] to denote the result of placing *t* in the hole of $C[\,]$.

A *position* *p* in a term *t* is represented by a finite sequence of natural numbers, where $\epsilon$ denotes the root position. We let $t|_p$ denote the *subterm* of *t* at position *p*, and $t[s]_p$ the result of replacing the subterm $t|_p$ by the term *s*. We denote by $s\trianglerighteq t$ that *t* is a subterm of *s*, and by $s\vartriangleright t$ that it is a *proper* subterm.

We write $\mathsf {Var}(t)$ to denote the set of variables appearing in a term *t*. A *substitution* is a mapping $\sigma : \mathcal{V}\rightarrow \mathcal{T}(\mathcal{F},\mathcal{V})$, which is extended to a morphism from $\mathcal{T}(\mathcal{F},\mathcal{V})$ to $\mathcal{T}(\mathcal{F},\mathcal{V})$ in the natural way. We denote the application of a substitution $\sigma$ to a term *t* by $t\sigma$.

A *rewrite rule* $l \rightarrow r$ is a pair of terms such that $l \notin \mathcal{V}$ and $\mathsf {Var}(l) \supseteq \mathsf {Var}(r)$. The terms *l* and *r* are called the left-hand side and the right-hand side of the rule, respectively. A *term rewrite system (TRS)* is a set of rewrite rules. Given a TRS $\mathcal{R}$, we write $\mathcal{F}_\mathcal{R}$ for the set of function symbols appearing in $\mathcal{R}$, $\mathcal{D}_\mathcal{R}$ for the set of the *defined* symbols, i.e., the root symbols of the left-hand sides of the rules, and $\mathcal{C}_\mathcal{R}$ for the set of *constructors*; $\mathcal{C}_\mathcal{R}= \mathcal{F}_\mathcal{R}\setminus \mathcal{D}_\mathcal{R}$.

For a TRS $\mathcal{R}$, we define the associated *rewrite relation*  as follows: given terms $s,t\in \mathcal{T}(\mathcal{F},\mathcal{V})$,  holds iff there exist a position *p* in *s*, a rewrite rule $l \rightarrow r \in \mathcal{R}$ and a substitution $\sigma$ with $s|_p = l\sigma$ and $t = s[r\sigma ]_p$; the rewrite step is often denoted by  to make the rewritten position explicit, and  if the position is strictly below the root.

Given a binary relation *R*, we denote its transitive closure by $R^{+}$ and reflexive transitive closure by $R^{*}$. We use the following generalized notion of orders.

### **Definition 1**

(*order pair*) We say that $(\succsim ,\succ )$ is a (well-founded) order pair on carrier *A* if $\;\succsim$ is a quasi-order on *A*, $\succ$ is a (well-founded) strict order on *A*, and they are compatible, i.e., ${\succsim } \circ {\succ } \circ {\succsim } \subseteq {\succ }$.

### The Dependency Pair Framework

The dependency pair (DP) method [2, 13] and its successor, the DP framework [12], enables analyzing cyclic dependencies between rewrite rules, and has become one of the most popular approaches to proving termination in term rewriting. Indeed, it underlies virtually all modern termination tools for TRSs.

Let us briefly recall the fundamentals of the DP framework. Here, we consider that the signature $\mathcal{F}$ is implicitly extended with fresh function symbols $f^\sharp$ for each defined function $f\in \mathcal{D}_\mathcal{R}$. Also, given a term $t = f(t_1,\ldots ,t_n)$ with $f\in \mathcal{D}_\mathcal{R}$, we let $t^\sharp$ denote $f^\sharp (t_1,\ldots ,t_n)$. If $l \rightarrow r \in \mathcal{R}$ and *t* is a subterm of *r* with a defined root symbol, then the rule $l^\sharp \rightarrow t^\sharp$ is a *dependency pair* of $\mathcal{R}$. The set of all dependency pairs of $\mathcal{R}$ is denoted by $\mathsf {DP}(\mathcal{R})$. Note that $\mathsf {DP}(\mathcal{R})$ is also a TRS.

A key ingredient in the DP framework is the notion of a *DP problem*, which is a pair $(\mathcal{P},\mathcal{R})$ of a set $\mathcal{P}$ of dependency pairs and a TRS $\mathcal{R}$. The DP problem $(\mathcal{P},\mathcal{R})$ is called *finite* if there is no infinite $(\mathcal{P},\mathcal{R})$-*chain*: A $(\mathcal{P},\mathcal{R})$-*chain* (*à la* [13]) is a possibly infinite rewrite sequence of the form:which informally represents a sequence of calls that can occur during a reduction. Then the basic result of Arts and Giesl [2] is formulated as follows:

#### **Proposition 1**

([2, 12]) A TRS $\mathcal{R}$ is terminating iff the DP problem $(\mathsf {DP}(\mathcal{R}),\mathcal{R})$ is finite.

To prove DP problems finite, various techniques have been proposed and implemented in current termination tools, e.g., AProVE, , NaTT, and so on. Among them, *reduction pairs* [2] constitute a fundamental technique: A *reduction pair* is a well-founded order pair $({\gtrsim },{>})$ on terms such that $\gtrsim$ is closed under contexts and substitutions, and $>$ is closed under substitutions.

#### **Proposition 2**

([2, 12, 13]) Let $(\mathcal{P},\mathcal{R})$ be a DP problem and $({\gtrsim },{>})$ a reduction pair such that $\mathcal{P}\cup \mathcal{R}\subseteq {\gtrsim }$. The DP problem $(\mathcal{P},\mathcal{R})$ is finite iff $(\mathcal{P}\setminus {>},\mathcal{R})$ is.

### Relative Termination

Given two relations *R* and *B*, we denote the relation $B^{*} \cdot R \cdot B^{*}$ by *R* / *B*. In particular, for two TRSs $\mathcal{R}$ and $\mathcal{B}$, we denote ${\rightarrow _\mathcal{R}}/{\rightarrow _\mathcal{B}}$ by .

Let us now recall the formal definition of relative termination:

#### **Definition 2**

(*relative termination* [21]) Let $\mathcal{R}$ and $\mathcal{B}$ be TRSs. We say that $\mathcal{R}$ *relatively terminates w.r.t.* $\mathcal{B}$, or simply that $\mathcal{R}/\mathcal{B}$ is terminating, if the relation  is terminating. We say that a term *t* is ${\mathcal{R}/\mathcal{B}}$-*nonterminating* if it starts an infinite  derivation, and ${\mathcal{R}/\mathcal{B}}$-*terminating* otherwise.

In other words, $\mathcal{R}/\mathcal{B}$ is terminating if every (possibly infinite) $\rightarrow _{\mathcal{R}\cup \mathcal{B}}$ derivation contains only finitely many $\rightarrow _{\mathcal{R}}$ steps. Note that sequences of $\rightarrow _{\mathcal{B}}$ steps are “collapsed” and seen as a single  step. Hence, an infinite  derivation must contain an infinite number of $\rightarrow _{\mathcal{R}}$ steps and thus only finite $\rightarrow _{\mathcal{B}}$ subderivations.

Geser [9] proposed a technique to reduce relative termination of TRSs to relative termination of simpler TRSs. This technique is later reformulated in the DP framework for proving standard termination, as *rule removal processors* [12]. We say a reduction pair $({\gtrsim },{>})$ is *monotone* if $>$ is closed under contexts.

#### **Proposition 3**

(relative rule removal processor) Let $\mathcal{R}$ and $\mathcal{B}$ be TRSs, and $({\gtrsim },{>})$ a monotone reduction pair such that $\mathcal{R}\cup \mathcal{B}\subseteq {\gtrsim }$. Then $\mathcal{R}$ is relatively terminating w.r.t. $\mathcal{B}$ if and only if $\mathcal{R}\setminus {>}$ is relatively terminating w.r.t. $\mathcal{B}\setminus {>}$.

## Relative Termination as a Dependency Pair Problem

The main goal of this paper is to introduce a counterpart of Proposition 1 for proving relative termination. In this section, we present a general approach that depends on the existence of a *proof ordering* 4 (see below) for the given TRSs. In the next section, we will focus on providing syntactic conditions that guarantee the existence of such a proof ordering.

Let us start with some basic conditions for relative termination in terms of DP problems. First, it is folklore that, given two TRSs $\mathcal{R}$ and $\mathcal{B}$, termination of $\mathcal{R}\cup \mathcal{B}$ implies termination of $\mathcal{R}/\mathcal{B}$. Therefore, an obvious *sufficient* condition for relative termination can be stated as follows:

### **Proposition 4**

For TRSs $\mathcal{R}$ and $\mathcal{B}$, $\mathcal{R}/\mathcal{B}$ is terminating if the DP problem $({\mathsf {DP}}(\mathcal{R}\cup \mathcal{B}),\mathcal{R}\cup \mathcal{B})$ is finite.

Observe that ${\mathsf {DP}}(\mathcal{R})\cup {\mathsf {DP}}(\mathcal{B})\subseteq {\mathsf {DP}}(\mathcal{R}\cup \mathcal{B})$ always holds, but ${\mathsf {DP}}(\mathcal{R}\cup \mathcal{B}) = {\mathsf {DP}}(\mathcal{R})\cup {\mathsf {DP}}(\mathcal{B})$ is not always true when there are shared symbols.

On the other hand, using a proof technique from the standard DP framework, we can easily prove the following *necessary* condition for relative termination.

### **Proposition 5**

Let $\mathcal{R}$ and $\mathcal{B}$ be TRSs. If $\mathcal{R}/\mathcal{B}$ is terminating, then the DP problem $({\mathsf {DP}}(\mathcal{R}),\mathrel {}\mathcal{R}\cup \mathcal{B})$ is finite.

### *Proof*

We prove that any infinite $(\mathsf {DP}(\mathcal{R}), \mathcal{R}\cup \mathcal{B})$-chain corresponds to an infinite  derivation. Consider a chainFor every $i = 1, 2, \dots$, there is a dependency pair $l_i^\sharp \rightarrow u_i^\sharp \in \mathsf {DP}(\mathcal{R})$ and a substitution $\sigma _i$ such that $s_i = l_i\sigma _i$ and $t_i = u_i\sigma _i$. As every dependency pair $l_i \rightarrow u_i$ corresponds to a rule $l_i \rightarrow r_i[u_i]_{p_i}$ in $\mathcal{R}$, we can construct the derivationwhich is an infinite  derivation. This concludes the proof. $\square$

Now, we aim at finding more precise characterizations of relative termination in terms of DP problems. We introduce the multisets that we will use to prove our main results.

### **Definition 3**

Let $\mathcal{R}$ be a TRS and *t* a term. The multiset $\triangledown _{\mathcal{R}}(t)$ of *maximal* $\mathcal{R}$-*defined subterms* of *t* is defined as follows:

- $$\triangledown _{\mathcal{R}}(x) = \emptyset $$ if $$x \in \mathcal{V}$$,
- $$\triangledown _{\mathcal{R}}(f(t_1,\dots ,t_n)) = \triangledown _{\mathcal{R}}(t_1) \cup \cdots \cup \triangledown _{\mathcal{R}}(t_n)$$ if $$f \notin \mathcal{D}_\mathcal{R}$$, and
- $$\triangledown _{\mathcal{R}}(f(t_1,\dots ,t_n)) = \{ f(t_1,\dots ,t_n) \}$$ if $$f \in \mathcal{D}_\mathcal{R}$$.

It is well known that a well-founded order pair can be extended to a well-founded order pair over multisets [32].

### **Definition 4**

(*multiset extension*) The *multiset extension* of an order pair $({\succsim },{\succ })$ on *A* is the order pair $({\succsim ^\mathrm {mul}},\mathrel {}{\succ ^\mathrm {mul}})$ on multisets over *A* which is defined as follows: $X \succsim ^\mathrm {mul}Y$ if *X* and *Y* are written $X = X^{\prime } \cup \{x_1,\ldots ,x_n\}$ and $Y = Y^{\prime } \cup \{y_1,\ldots ,y_n\}$ such that

- $$\forall y \in Y^{\prime }.\ \exists x \in X^{\prime }.\ x \succ y$$, and
- $$\forall i \in \{1,\ldots ,n\}.\ x_i \succsim y_i$$.

We have $X \succ ^\mathrm {mul}Y$ if it also holds that $X^{\prime } \ne \emptyset$.

Our main result in this section requires a particular order pair that satisfies the following property:

### **Definition 5**

Let $\mathcal{R}$ and $\mathcal{B}$ be TRSs. We say that a pair $(\succsim ,\succ )$ of relations is a *proof ordering* for $\mathcal{R}/\mathcal{B}$, if it is a well-founded order pair on $\mathcal{R}/\mathcal{B}$-terminating terms, and satisfies the following assumptions:

### **Assumption 1**

$$s \rightarrow _\mathcal{R}t$$ implies $$\triangledown _{\mathcal{R}}(s) \succ ^\mathrm {mul}\triangledown _{\mathcal{R}}(t)$$, and

### **Assumption 2**

$$s \rightarrow _\mathcal{B}t$$ implies $$\triangledown _{\mathcal{R}}(s) \succsim ^\mathrm {mul}\triangledown _{\mathcal{R}}(t)$$.

In the following, we say that $\mathcal{R}$ and $\mathcal{B}$ *admits a proof ordering* if there exists at least one proof ordering for $\mathcal{R}/\mathcal{B}$.

A natural question is whether such a proof ordering exists for some given TRSs $\mathcal{R}$ and $\mathcal{B}$. We will investigate this question in the next section.

Now, we can show the following auxiliary result, which is an easy consequence of the fact that the multiset extension preserves well-foundedness.

### **Lemma 1**

Let $\mathcal{R}$ and $\mathcal{B}$ be TRSs that admit a proof ordering. A term *s* is ${\mathcal{R}/\mathcal{B}}$-terminating if all elements in $\triangledown _{\mathcal{R}}(s)$ are ${\mathcal{R}/\mathcal{B}}$-terminating.

### *Proof*

We prove the claim by contradiction. Assume that all elements in $\triangledown _{\mathcal{R}}(s)$ are ${\mathcal{R}/\mathcal{B}}$-terminating but *s* is not ${\mathcal{R}/\mathcal{B}}$-terminating. Hence there exists an infinite sequence of the following form:1Using Assumptions 1 and 2, we obtain the following sequence for the proof ordering $(\succsim ,\succ )$:

$$\begin{aligned} \triangledown _{\mathcal{R}}(s) \succsim ^\mathrm {mul}\triangledown _{\mathcal{R}}(t_1) \succ ^\mathrm {mul}\triangledown _{\mathcal{R}}(t_1^{\prime }) \succsim ^\mathrm {mul}\triangledown _{\mathcal{R}}(t_2) \succ ^\mathrm {mul}\triangledown _{\mathcal{R}}(t_2^{\prime }) \succsim ^\mathrm {mul}\cdots \end{aligned}$$

According to Thiemann et al. [32, Section 3] and the assumption that $(\succsim ,\succ )$ is a well-founded order pair on ${\mathcal{R}/\mathcal{B}}$-terminating terms, the multiset extension $({\succsim ^\mathrm {mul}},{\succ ^\mathrm {mul}})$ is a well-founded order pair on multisets of ${\mathcal{R}/\mathcal{B}}$-terminating terms. Hence, $\triangledown _{\mathcal{R}}(s)$ cannot start such an infinite $\succ ^\mathrm {mul}$-reduction sequence, which contradicts our assumption. Hence the infinite sequence (1) cannot exist and thus *s* is ${\mathcal{R}/\mathcal{B}}$-terminating. $\square$

Following the common practice in the termination literature, we say that an ${\mathcal{R}/\mathcal{B}}$-nonterminating term is *minimal* if all its proper subterms are ${\mathcal{R}/\mathcal{B}}$-terminating. It is clear that any ${\mathcal{R}/\mathcal{B}}$-nonterminating term has some minimal ${\mathcal{R}/\mathcal{B}}$-nonterminating subterm. The following lemma proves an essential result in our approach.

### **Lemma 2**

Let $\mathcal{R}$ and $\mathcal{B}$ be TRSs that admit a proof ordering. If *t* is a minimal $\mathcal{R}/\mathcal{B}$-nonterminating term, then $\mathsf {root}(t)\in \mathcal{D}_\mathcal{R}$.

### *Proof*

We prove the claim by contradiction. Consider a minimal $\mathcal{R}/\mathcal{B}$-nonterminating term *s* such that $\mathsf {root}(s) \notin \mathcal{D}_\mathcal{R}$. Since $\mathsf {root}(s) \notin \mathcal{D}_\mathcal{R}$, all elements in $\triangledown _{\mathcal{R}}(s)$ are proper subterms of *s*, which are $\mathcal{R}/\mathcal{B}$-terminating due to minimality. Lemma 1 implies that *s* is $\mathcal{R}/\mathcal{B}$-terminating, and hence we have a contradiction. $\square$

Using the previous result, we can state the following sufficient condition which states that termination of $\mathcal{R}/\mathcal{B}$ coincides with the finiteness of the DP problem $(\mathsf {DP}(\mathcal{R}), \mathcal{R}\cup \mathcal{B})$, even if $\mathcal{B}$ is non-terminating. Here, we further impose that $\mathcal{R}$ and $\mathcal{B}$ share no defined symbol, i.e., $\mathcal{D}_\mathcal{R}\cap \mathcal{D}_\mathcal{B}= \emptyset$.

### **Theorem 1**

Let $\mathcal{R}$ and $\mathcal{B}$ be TRSs that admit a proof ordering. Let $\mathcal{D}_\mathcal{R}\cap \mathcal{D}_\mathcal{B}= \emptyset$. Then, $\mathcal{R}/\mathcal{B}$ is terminating iff the DP problem $(\mathsf {DP}(\mathcal{R}), \mathcal{R}\cup \mathcal{B})$ is finite.

### *Proof*

The “only-if” direction follows from Proposition 5. We show the “if” direction by constructing an infinite chain from an arbitrary infinite  sequence:W.l.o.g., we consider $s_1$ to be minimal. By Lemma 2, we have $\mathsf {root}(s_1) \in \mathcal{D}_\mathcal{R}$. Due to the assumption $\mathcal{D}_\mathcal{R}\cap \mathcal{D}_\mathcal{B}= \emptyset$, a $\rightarrow _\mathcal{B}$-reduction cannot occur at the root position unless an $\rightarrow _\mathcal{R}$-reduction rewrites the root symbol. Thus, due to minimality, there exists some *n* such thatLet $t_n = l\sigma$ and $s_{n+1} = r\sigma$ for $l \rightarrow r \in \mathcal{R}$, and consider a minimal ${\mathcal{R}/\mathcal{B}}$-nonterminating subterm *u* of $s_{n+1}$. Here, $t_n$ is minimal since $s_1$ is. Thus, there must exist a non-variable subterm *v* of *r* such that $u = v\sigma$. Again by Lemma 2, we have $\mathsf {root}(v) \in \mathcal{D}_\mathcal{R}$ and thus $l^\sharp \rightarrow v^\sharp \in \mathsf {DP}(\mathcal{R})$. We obtainContinuing the construction from *u*, we obtain an infinite $(\mathsf {DP}(\mathcal{R}),\mathcal{R}\cup \mathcal{B})$-chain. $\square$

The condition $\mathcal{D}_\mathcal{R}\cap \mathcal{D}_\mathcal{B}= \emptyset$ will be lifted in Sect. 5. However, at this point, it is necessary as the following example illustrates:

### *Example 1*

Consider the following TRSs:

$$\begin{aligned} \mathcal{R}&= \{\,\mathsf {f}(x) \rightarrow x\,\}&\mathcal{B}&= \{\,\mathsf {f}(x) \rightarrow \mathsf {f}(\mathsf {f}(x))\,\} \end{aligned}$$

with $\mathcal{D}_\mathcal{R}\cap \mathcal{D}_\mathcal{B}\ne \emptyset$. Here, we can define a proof ordering $(\succ ,\succsim )$ for $\mathcal{R}/\mathcal{B}$ as follows: ${\succ } = {\rightarrow _{\mathcal{R}/\mathcal{B}}^+}$ and ${\succsim } = {\rightarrow _\mathcal{B}^{*}}$; note that $\succ$ is well-founded on $\mathcal{R}/\mathcal{B}$-terminating terms, i.e., variables. Here, the DP problem $(\mathsf {DP}(\mathcal{R}),\mathcal{R}\cup \mathcal{B})$ is trivially finite as $\mathsf {DP}(\mathcal{R}) = \emptyset$, but $\mathcal{R}/\mathcal{B}$ is not terminating (consider, e.g., the infinite derivation ${\mathsf {f}}(x) \rightarrow _\mathcal{B}{\mathsf {f}}({\mathsf {f}}(x)) \rightarrow _\mathcal{R}{\mathsf {f}}(x) \rightarrow _\mathcal{B}\cdots$).

We note that, to make Theorem 1 applicable in practice, we must find a feasible way to check if $\mathcal{R}$ and $\mathcal{B}$ admit a proof ordering. In the next section, we introduce syntactic conditions on $\mathcal{R}$ and $\mathcal{B}$ for this purpose.

## Syntactic Conditions for Admitting a Proof Ordering

In this section, given TRSs $\mathcal{R}$ and $\mathcal{B}$, we focus on providing syntactic conditions on $\mathcal{R}$ and $\mathcal{B}$ such that they admit a proof ordering.

First, we consider satisfying Assumption 1. To this goal, we have the following simple sufficient condition:

### **Lemma 3**

Let $\mathcal{R}$ be a TRS and $(\succsim ,\succ )$ an order pair.^5^ If ${\rightarrow _\mathcal{R}} \cdot {\trianglerighteq } \subseteq {\succ }$, then $s \rightarrow _\mathcal{R}t$ implies $\triangledown _{\mathcal{R}}(s) \succ ^\mathrm {mul}\triangledown _{\mathcal{R}}(t)$.

### *Proof*

Let  and *q* be the shortest prefix of *p* such that $\mathsf {root}(s|_q) \in \mathcal{D}_\mathcal{R}$, that is, $\triangledown _{\mathcal{R}}(s) = \triangledown _{\mathcal{R}}(s[\,]_q) \cup \{ s|_q \}$. Note that *q* always exists since $\mathsf {root}(s|_p) \in \mathcal{D}_\mathcal{R}$. We distinguish the following cases:

- Assume that $q < p$. Since $\mathsf {root}(s|_q) = \mathsf {root}(t|_q) \in \mathcal{D}_\mathcal{R}$, we have $\triangledown _{\mathcal{R}}(t) = \triangledown _{\mathcal{R}}(s[\,]_q) \cup \{ t|_q \}$. From $s \rightarrow _\mathcal{R}t$ we get $s|_q \succ t|_q$ by the assumption, and thus $\triangledown _{\mathcal{R}}(s) \succ ^\mathrm {mul}\triangledown _{\mathcal{R}}(t)$.
- Assume that $p = q$. We have $\triangledown _{\mathcal{R}}(t) = \triangledown _{\mathcal{R}}(s[\,]_p) \cup \triangledown _{\mathcal{R}}(t|_p)$. For every $t^{\prime } \in \triangledown _{\mathcal{R}}(t|_p)$, we have $s|_p \rightarrow _\mathcal{R}t|_p \trianglerighteq t^{\prime }$ and thus $s|_p \succ t^{\prime }$ by the assumption. We conclude $\triangledown _{\mathcal{R}}(s) \succ ^\mathrm {mul}\triangledown _{\mathcal{R}}(t)$.$\square$

On the other hand, satisfying Assumption 2 is not that easy. For this purpose, we require $\mathcal{B}$ to be *non-duplicating*, i.e., no variable has more occurrences in the right-hand side of a rule than in the left-hand side, together with the following condition:

### **Definition 6**

(*dominance*) We say that a TRS $\mathcal{R}$ *dominates* a TRS $\mathcal{B}$ iff the right-hand sides of all rules in $\mathcal{B}$ contain no symbol from $\mathcal{D}_\mathcal{R}$.

Under these assumptions, we will show that the following definition yields indeed a proof ordering for $\mathcal{R}/\mathcal{B}$.

### **Definition 7**

For two TRSs $\mathcal{R}$ and $\mathcal{B}$, the pair $(\succsim _{\mathcal{R}/\mathcal{B}},\succ _{\mathcal{R}/\mathcal{B}})$ of relations on terms is defined as follows: ${\succsim }_{\mathcal{R}/\mathcal{B}} = ({\vartriangleright _\mathcal{R}} \cup {\rightarrow _\mathcal{B}})^{*}$ and ${\succ }_{\mathcal{R}/\mathcal{B}} = ({\vartriangleright _\mathcal{R}}/{\rightarrow _\mathcal{B}})^+$. Here, $\vartriangleright _\mathcal{R}$ is defined as follows: $s \vartriangleright _\mathcal{R}t$ iff $s \rightarrow _\mathcal{R}\cdot \trianglerighteq t$, or $s \vartriangleright t$ with $\mathsf {root}(s) \in \mathcal{D}_\mathcal{R}$.

The relations $\succsim _{\mathcal{R}/\mathcal{B}}$ and $\succ _{\mathcal{R}/\mathcal{B}}$ enjoy the following key property:

### **Lemma 4**

Let $\mathcal{R}$ and $\mathcal{B}$ be TRSs such that $\mathcal{B}$ is non-duplicating and $\mathcal{R}$ dominates $\mathcal{B}$. Then $(\succsim _{\mathcal{R}/\mathcal{B}},\succ _{\mathcal{R}/\mathcal{B}})$ is a well-founded order pair on ${\mathcal{R}/\mathcal{B}}$-terminating terms.

### *Proof*

Trivially $\succsim _{\mathcal{R}/\mathcal{B}}$ is a quasi-order, and $\succ _{\mathcal{R}/\mathcal{B}}$ is transitive. Compatibility is also clear from the definition. The remaining goal is to prove that $\succ _{\mathcal{R}/\mathcal{B}}$ is well-founded on ${\mathcal{R}/\mathcal{B}}$-terminating terms. To this end, suppose on the contrary that there exists an infinite $\succ _{\mathcal{R}/\mathcal{B}}$-sequence, that is,

2 $$\begin{aligned} s_1 \vartriangleright _\mathcal{R}t_1 \rightarrow _\mathcal{B}^{*} s_2 \vartriangleright _\mathcal{R}t_2 \rightarrow _\mathcal{B}^{*} \cdots \end{aligned}$$

starting from ${\mathcal{R}/\mathcal{B}}$-terminating term $s_1$. Note that (2) is actually an infinite $({\rightarrow _\mathcal{R}} \cup {\rightarrow _\mathcal{B}} \cup {\vartriangleright })$-sequence. Since $s_1$ is $\mathcal{R}/\mathcal{B}$-terminating, (2) cannot contain infinitely many $\rightarrow _\mathcal{R}$-steps. Hence there must be some *k* such that

$$\begin{aligned} s_k \vartriangleright t_k \rightarrow _\mathcal{B}^{*} s_{k+1} \vartriangleright t_{k+1} \rightarrow _\mathcal{B}^{*} \cdots \end{aligned}$$

where $\mathsf {root}(s_i) \in \mathcal{D}_\mathcal{R}$ for each $i = k, k+1, \dots$. Thus, the number of $\mathcal{D}_\mathcal{R}$ symbols strictly decreases in each $\vartriangleright$ step. On the other hand, the non-duplication and dominance conditions entails that the number does not increase by $\rightarrow _\mathcal{B}$ steps. Hence, the sequence must terminate and concludes the desired well-foundedness. $\square$

Now, in order to prove that $\succsim _{\mathcal{R}/\mathcal{B}}$ satisfies Assumption 2, we need the following auxiliary result, where $\mathsf {MVar}(s)$ denotes the multiset of variables occurring in a term *s*.

### **Lemma 5**

Let $\mathcal{R}$ and $\mathcal{B}$ be TRSs. For every term *t* and substitution $\sigma$, $\triangledown _{\mathcal{R}}(t\sigma ) \succsim _{\mathcal{R}/\mathcal{B}}^\mathrm {mul}\bigcup _{x \in \mathsf {MVar}(t)} \triangledown _{\mathcal{R}}(x\sigma )$.

### *Proof*

We prove the claim by structural induction on *t*. It is trivial if *t* is a variable. Hence, we assume $t = f(t_1,\dots ,t_n)$. We proceed by case analysis on whether $f \in \mathcal{D}_\mathcal{R}$ or not.

- If $f \in \mathcal{D}_\mathcal{R}$, then we have $\triangledown _{\mathcal{R}}(t\sigma ) = \{t\sigma \}$. For every $x \in \mathsf {MVar}(t)$, we have $t\sigma \vartriangleright _\mathcal{R}x\sigma$ and hence $t\sigma \succ _{\mathcal{R}/\mathcal{B}} x\sigma$. Thus we conclude the claim.
- If $f \notin \mathcal{D}_\mathcal{R}$, then we have $\triangledown _{\mathcal{R}}(t\sigma ) = \triangledown _{\mathcal{R}}(t_1\sigma ) \cup \cdots \cup \triangledown _{\mathcal{R}}(t_n\sigma )$. Applying the induction hypothesis, we obtain  Note that $\mathsf {MVar}(t) = \mathsf {MVar}(t_1) \cup \cdots \cup \mathsf {MVar}(t_n)$.$\square$

The following lemma is the key result of this section:

### **Lemma 6**

Let $\mathcal{R}$ and $\mathcal{B}$ be TRSs such that $\mathcal{B}$ is non-duplicating and $\mathcal{R}$ dominates $\mathcal{B}$. If $s \rightarrow _\mathcal{B}t$, then $\triangledown _{\mathcal{R}}(s) \succsim _{\mathcal{R}/\mathcal{B}}^\mathrm {mul}\triangledown _{\mathcal{R}}(t)$.

### *Proof*

We prove that  implies $\triangledown _{\mathcal{R}}(s) \succsim _{\mathcal{R}/\mathcal{B}}^\mathrm {mul}\triangledown _{\mathcal{R}}(t)$ for arbitrary terms *s* and *t* and a position *p*. We distinguish the following two cases:

- First, assume that *p* has a proper prefix *q* such that $s|_q \in \triangledown _{\mathcal{R}}(s)$. We have
  $$\begin{aligned} \triangledown _{\mathcal{R}}(s) = \triangledown _{\mathcal{R}}(s[\,]_q) \cup \{ s|_q \}&\text {and}\quad \triangledown _{\mathcal{R}}(t) = \triangledown _{\mathcal{R}}(s[\,]_q) \cup \{ t|_q \} \end{aligned}$$
  Since $s|_q \rightarrow _\mathcal{B}t|_q$, we have $s|_q \succsim _{\mathcal{R}/\mathcal{B}} t|_q$ and thus $\triangledown _{\mathcal{R}}(s) \succsim _{\mathcal{R}/\mathcal{B}}^\mathrm {mul}\triangledown _{\mathcal{R}}(t)$.
- Assume now that $s_q \notin \triangledown _{\mathcal{R}}(s)$ for any proper prefix *q* of *p*. Let $l \rightarrow r \in \mathcal{B}$, $s|_p = l\sigma$, and $t = s[r\sigma ]_p$. In this case, we have
  $$\begin{aligned} \triangledown _{\mathcal{R}}(s) = \triangledown _{\mathcal{R}}(s[\,]_p) \cup \triangledown _{\mathcal{R}}(l\sigma )&\text {and}\quad \triangledown _{\mathcal{R}}(t) = \triangledown _{\mathcal{R}}(s[\,]_p) \cup \triangledown _{\mathcal{R}}(r\sigma ) \end{aligned}$$
  From Lemma 5, we have $\triangledown _{\mathcal{R}}(l\sigma ) \succsim _{\mathcal{R}/\mathcal{B}}^\mathrm {mul}\bigcup _{x \in \mathsf {MVar}(l)} \triangledown _{\mathcal{R}}(x\sigma )$. Since $\mathcal{R}$ dominates $\mathcal{B}$, *r* cannot contain symbols from $\mathcal{D}_\mathcal{R}$. Therefore,
  $$\begin{aligned} \triangledown _{\mathcal{R}}(l\sigma ) \succsim _{\mathcal{R}/\mathcal{B}}^\mathrm {mul}\textstyle \bigcup _{x \in \mathsf {MVar}(l)} \triangledown _{\mathcal{R}}(x\sigma )&\text {and}\quad \triangledown _{\mathcal{R}}(r\sigma ) = \textstyle \bigcup _{x \in \mathsf {MVar}(r)} \triangledown _{\mathcal{R}}(x\sigma ) \end{aligned}$$
  Since $\mathcal{B}$ is non-duplicating, we have $\mathsf {MVar}(l) \supseteq \mathsf {MVar}(r)$ and thus $\triangledown _{\mathcal{R}}(l\sigma ) \supseteq \triangledown _{\mathcal{R}}(r\sigma )$. Therefore, we conclude $\triangledown _{\mathcal{R}}(s) \succsim _{\mathcal{R}/\mathcal{B}}^\mathrm {mul}\triangledown _{\mathcal{R}}(t)$.$\square$

Finally, since the premise of Lemma 3 trivially holds for the order pair of Definition 7, the following result is a direct consequence of Theorem 1, Lemma 3, and Lemma 6.

### **Corollary 1**

Let $\mathcal{R}$ and $\mathcal{B}$ be TRSs such that $\mathcal{B}$ is non-duplicating, $\mathcal{R}$ dominates $\mathcal{B}$, and $\mathcal{D}_\mathcal{R}\cap \mathcal{D}_\mathcal{B}= \emptyset$. Then, $\mathcal{R}/\mathcal{B}$ is terminating iff the DP problem $(\mathsf {DP}(\mathcal{R}), \mathcal{R}\cup \mathcal{B})$ is finite.

The following simple example illustrates that Corollary 1 indeed advances the state of the art in proving relative termination.

### *Example 2*

Consider the following two TRSs:

$$\begin{aligned} \mathcal{R}= \{~ {\mathsf {g}}({\mathsf {s}}(x),y) \rightarrow {\mathsf {g}}({\mathsf {f}}(x,y),y) ~\} ~~~~ \mathcal{B}= \{~ {\mathsf {f}}(x,y) \rightarrow x, ~ {\mathsf {f}}(x,y) \rightarrow {\mathsf {f}}(x,{\mathsf {s}}(y)) ~\} \end{aligned}$$

Since they satisfy the conditions of Corollary 1, we obtain the DP problem $(\mathsf {DP}(\mathcal{R}),\mathrel {}\mathcal{R}\cup \mathcal{B})$, where $\mathsf {DP}(\mathcal{R}) = \{\,\mathsf {g}^\sharp (\mathsf {s}(x),y) \rightarrow \mathsf {g}^\sharp (\mathsf {f}(x,y),y)\,\}$. The DP problem can be proved finite using classic techniques, e.g., polynomial interpretation ${\mathcal {P} ol }$ such that ${\mathsf {f}}_{\mathcal {P} ol }(x,y) = x$. On the other hand, all the previous tools we know that support relative termination, namely AProVE (ver 2014),  (ver 1.15), Jambox (ver 2006) [8], and TPA (ver 1.1) [22], fail on this problem.

The dominance condition and the non-duplication condition are indeed necessary for Corollary 1 to hold. It is easy to see that the former condition is necessary:

### *Example 3*

Consider the two TRSs $\mathcal{R}= \{\,\mathsf {a} \rightarrow \mathsf {b}\,\}$ and $\mathcal{B}= \{\,\mathsf {b} \rightarrow \mathsf {a}\,\}$, which violates the dominance condition. Obviously, $\mathcal{R}/\mathcal{B}$ is not terminating. However, $(\mathsf {DP}(\mathcal{R}),\mathcal{R}\cup \mathcal{B})$ is trivially finite since $\mathsf {DP}(\mathcal{R}) = \emptyset$.

The following example illustrates that the latter condition is also necessary:

### *Example 4*

Consider the following two TRSs:

$$\begin{aligned} \mathcal{R}&= \{\ \mathsf {a} \rightarrow \mathsf {b}\ \}&\mathcal{B}&= \{\ {\mathsf {f}}(x) \rightarrow \mathsf {c}(x,{\mathsf {f}}(x))\ \} \end{aligned}$$

which violates the non-duplication condition. The DP problem $(\mathsf {DP}(\mathcal{R}),\mathcal{R}\cup \mathcal{B})$ is trivially finite since $\mathsf {DP}(\mathcal{R})=\emptyset$. However, we have the following infinite -derivation:

$$\begin{aligned} {\mathsf {f}}(\mathsf {a}) \rightarrow _\mathcal{B}\mathsf {c}(\mathsf {a},{\mathsf {f}}(\mathsf {a})) \rightarrow _\mathcal{R}\mathsf {c}(\mathsf {b},{\mathsf {f}}(\mathsf {a})) \rightarrow _\mathcal{B}\mathsf {c}(\mathsf {b},\mathsf {c}(\mathsf {a},{\mathsf {f}}(\mathsf {a}))) \rightarrow _\mathcal{R}\mathsf {c}(\mathsf {b},\mathsf {c}(\mathsf {b},{\mathsf {f}}(\mathsf {a}))) \rightarrow _\mathcal{B}\cdots \end{aligned}$$

Note that this is a counterexample against [18, Theorem 5].

## Improving Applicability

When dominance and non-duplication are assumed, the condition $\mathcal{D}_\mathcal{R}\cap \mathcal{D}_\mathcal{B}= \emptyset$ is not necessary anymore. In order to show that this is indeed the case, let us recall the following result by Geser [9]:

### **Proposition 6**

Let $\mathcal{R}$, $\mathcal{B}^{\prime }$, and $\mathcal{B}^{{\prime }{\prime }}$ be TRSs. Then, $(\mathcal{R}\cup \mathcal{B}^{\prime })/\mathcal{B}^{{\prime }{\prime }}$ is terminating iff both $\mathcal{R}/(\mathcal{B}^{\prime }\cup \mathcal{B}^{{\prime }{\prime }})$ and $\mathcal{B}^{\prime }/\mathcal{B}^{{\prime }{\prime }}$ are terminating.

The following result removes the condition $\mathcal{D}_\mathcal{R}\cap \mathcal{D}_\mathcal{B}= \emptyset$ from Corollary 1:

### **Theorem 2**

Let $\mathcal{R}$ and $\mathcal{B}$ be TRSs such that $\mathcal{B}$ is non-duplicating and $\mathcal{R}$ dominates $\mathcal{B}$. Then, $\mathcal{R}/\mathcal{B}$ is terminating iff the DP problem $(\mathsf {DP}(\mathcal{R}), \mathcal{R}\cup \mathcal{B})$ is finite.

### *Proof*

Let $\mathcal{B}^{\prime }$ be the set of rules in $\mathcal{B}$ that define $\mathcal{D}_\mathcal{R}$ symbols, i.e.,

$$\begin{aligned} \mathcal{B}^{\prime } = \{\, l \rightarrow r \in \mathcal{B}\mid \mathsf {root}(l) \in \mathcal{D}_\mathcal{R}\,\} \end{aligned}$$

and let $\mathcal{B}^{{\prime }{\prime }} = \mathcal{B}\setminus \mathcal{B}^{\prime }$. Due to the dominance condition, the right-hand sides of $\mathcal{B}^{\prime }$ rules cannot contain symbols from $\mathcal{D}_\mathcal{R}$ $(=\mathcal{D}_{\mathcal{R}\cup \mathcal{B}^{\prime }})$. Hence we have $\mathsf {DP}(\mathcal{B}^{\prime }) = \emptyset$ and $\mathsf {DP}(\mathcal{R}\cup \mathcal{B}^{\prime }) = \mathsf {DP}(\mathcal{R})$.

First, observe that $\mathcal{B}^{{\prime }{\prime }}$ is non-duplicating, $\mathcal{B}^{\prime }$ dominates $\mathcal{B}^{{\prime }{\prime }}$, and $\mathcal{D}_{\mathcal{B}^{\prime }} \cap \mathcal{D}_{\mathcal{B}^{{\prime }{\prime }}} = \emptyset$. Hence, Corollary 1 ensures the termination of $\mathcal{B}^{\prime }/\mathcal{B}^{{\prime }{\prime }}$ via finiteness of the DP problem $(\mathsf {DP}(\mathcal{B}^{\prime }),\mathcal{B})$, which is trivial since $\mathsf {DP}(\mathcal{B}^{\prime }) = \emptyset$. Hence by Proposition 6, $\mathcal{R}/\mathcal{B}$ is terminating iff $(\mathcal{R}\cup \mathcal{B}^{\prime })/\mathcal{B}^{{\prime }{\prime }}$ is terminating.

Next, observe that $\mathcal{B}^{{\prime }{\prime }}$ is non-duplicating, $\mathcal{R}\cup \mathcal{B}^{\prime }$ dominates $\mathcal{B}^{{\prime }{\prime }}$, and $\mathcal{D}_{\mathcal{R}\cup \mathcal{B}^{\prime }} \cap \mathcal{D}_{\mathcal{B}^{{\prime }{\prime }}} = \emptyset$. Hence by Corollary 1, $(\mathcal{R}\cup \mathcal{B}^{\prime })/\mathcal{B}^{{\prime }{\prime }}$ is terminating iff the DP problem $(\mathsf {DP}(\mathcal{R}),\mathcal{R}\cup \mathcal{B})$ is finite. $\square$

The remaining two conditions, namely the dominance and non-duplication conditions might be still too restrictive in practice. For instance, only six out of 44 examples in the relative TRS category of TPDB 9.0 satisfy both conditions.

Luckily, we can employ again Proposition 6 to relax the conditions, at the cost of losing completeness. More precisely, we use the following corollary of Proposition 6:

### **Corollary 2**

Let $\mathcal{R}$, $\mathcal{B}^{\prime }$, and $\mathcal{B}^{{\prime }{\prime }}$ be TRSs. If $(\mathcal{R}\cup \mathcal{B}^{\prime })/\mathcal{B}^{{\prime }{\prime }}$ is terminating, then $\mathcal{R}/(\mathcal{B}^{\prime }\cup \mathcal{B}^{{\prime }{\prime }})$ is terminating.

Consider TRSs $\mathcal{R}$ and $\mathcal{B}$, and that we want to prove termination of $\mathcal{R}/\mathcal{B}$ but the conditions of Theorem 2 do not hold. Then, we might still find a partition $\mathcal{B}= \mathcal{B}^{\prime }\uplus \mathcal{B}^{{\prime }{\prime }}$ such that $\mathcal{R}\cup \mathcal{B}^{\prime }$ and $\mathcal{B}^{{\prime }{\prime }}$ satisfy the conditions. If we succeed, then we can prove termination of $\mathcal{R}/\mathcal{B}$ as follows: First, we obtain termination of $(\mathcal{R}\cup \mathcal{B}^{\prime })/\mathcal{B}^{{\prime }{\prime }}$ by Theorem 2, and then that of $\mathcal{R}/(\mathcal{B}^{\prime }\cup \mathcal{B}^{{\prime }{\prime }})$, i.e., $\mathcal{R}/\mathcal{B}$ by Corollary 2.

### **Corollary 3**

Let $\mathcal{R}$ and $\mathcal{B}$ be TRSs. If $\mathcal{B}$ is split into $\mathcal{B}= \mathcal{B}^{\prime }\uplus \mathcal{B}^{{\prime }{\prime }}$ such that (1) $\mathcal{B}^{{\prime }{\prime }}$ is non-duplicating, (2) $\mathcal{R}\cup \mathcal{B}^{\prime }$ dominates $\mathcal{B}^{{\prime }{\prime }}$, and (3) the DP problem $(\mathsf {DP}(\mathcal{R}\cup \mathcal{B}^{\prime }),\mathcal{R}\cup \mathcal{B})$ is finite, then $\mathcal{R}/\mathcal{B}$ is terminating.

### *Example 5*

Consider the following TRSs $\mathcal{R}$ and $\mathcal{B}$:

$$\begin{aligned} \mathcal{R}&= \{\,\mathsf {a} \rightarrow \mathsf {b}\,\}&\mathcal{B}&= \{\, \mathsf {f}(\mathsf {s}(x)) \rightarrow \mathsf {c}(x,\mathsf {f}(x)),\ \mathsf {c}(x,\mathsf {c}(y,z)) \rightarrow \mathsf {c}(y,\mathsf {c}(x,z)) \,\} \end{aligned}$$

The first rule of $\mathcal{B}$ is duplicating, and hence Theorem 2 does not apply. However, we can split $\mathcal{B}$ into the following TRSs $\mathcal{B}^{\prime }$ and $\mathcal{B}^{{\prime }{\prime }}$:

$$\begin{aligned} \mathcal{B}^{\prime }&= \{\,\mathsf {f}(\mathsf {s}(x)) \rightarrow \mathsf {c}(x,\mathsf {f}(x))\,\}&\mathcal{B}^{{\prime }{\prime }}&= \{\,\mathsf {c}(x,\mathsf {c}(y,z)) \rightarrow \mathsf {c}(y,\mathsf {c}(x,z))\,\} \end{aligned}$$

so that Corollary 3 applies. Now, we have $\mathsf {DP}(\mathcal{R}\cup \mathcal{B}^{\prime }) = \{\,\mathsf {f}^\sharp (\mathsf {s}(x)) \rightarrow \mathsf {f}^\sharp (x)\,\}$, whose finiteness can be proved using standard techniques.

To show termination of $\mathcal{R}/\mathcal{B}$ by Corollary 3, two kinds of $\mathcal{B}$-rules must not be used infinitely many times, namely, duplicating ones (1), and those which violate the dominance condition (2). This is not overly restrictive; intuitively speaking, the rules of the first kind often (not always) *duplicate* an $\mathcal{R}$-redex infinitely many times, and those of the second kind often *generate* infinitely many $\mathcal{R}$-redexes.

### *Example 6*

Consider the following TRSs $\mathcal{R}$ and $\mathcal{B}$:

$$\begin{aligned} \mathcal{R}&= \{\, \mathsf {a^{\prime }} \rightarrow \mathsf {a^{{\prime }{\prime }}} \,\}&\mathcal{B}&= \{\, \mathsf {a} \rightarrow \mathsf {a^{\prime }},\,\mathsf {d} \rightarrow \mathsf {c}(\mathsf {a},\mathsf {d}) \,\} \end{aligned}$$

Note that $\mathcal{R}$ does not dominate $\mathcal{B}$. The DP problem $(\mathsf {DP}(\mathcal{R}),\mathrel {} \mathcal{R}\cup \mathcal{B})$ is trivially finite since $\mathsf {DP}(\mathcal{R}) = \emptyset$. However, $\mathcal{R}$ is not relatively terminating w.r.t. $\mathcal{B}$, as the following infinite derivation exists:

$$\begin{aligned} \begin{array}{llllll} \mathsf {d} &{} \rightarrow _\mathcal{B}&{} \mathsf {c}(\mathsf {a},\mathsf {d}) \rightarrow _\mathcal{B}\mathsf {c}(\mathsf {a^{\prime }},\mathsf {d}) \rightarrow _\mathcal{R}\mathsf {c}(\mathsf {a^{{\prime }{\prime }}},\mathsf {d}) \\ &{} \rightarrow _\mathcal{B}&{} \mathsf {c}(\mathsf {a^{{\prime }{\prime }}},\mathsf {c}(\mathsf {a},\mathsf {d})) \rightarrow _\mathcal{B}\mathsf {c}(\mathsf {a^{{\prime }{\prime }}},\mathsf {c}(\mathsf {a^{\prime }},\mathsf {d})) \rightarrow _\mathcal{R}\mathsf {c}(\mathsf {a^{{\prime }{\prime }}},\mathsf {c}(\mathsf {a^{{\prime }{\prime }}},\mathsf {d})) \rightarrow _\mathcal{B}\cdots \end{array} \end{aligned}$$

There is no partition of $\mathcal{B}$, i.e., $\mathcal{B}= \mathcal{B}^{\prime }\uplus \mathcal{B}^{{\prime }{\prime }}$ such that $\mathcal{R}\cup \mathcal{B}^{\prime }$ dominates $\mathcal{B}^{{\prime }{\prime }}$ and, thus, Corollary 3 is not applicable, as expected.

### *Example 7*

Consider an arbitrary nonempty TRS $\mathcal{R}$ together with a TRS $\mathcal{B}$ of the form $\mathcal{B}= \mathcal{B}^{\prime }\uplus \mathcal{B}^{{\prime }{\prime }}$ with $\mathcal{B}^{{\prime }{\prime }} = \{\ {\mathsf {f}}(x) \rightarrow \mathsf {c}(x,{\mathsf {f}}(x))\ \}$, which is duplicating and non-terminating (so Corollary 3 does not apply). Here, regardless of the rules of $\mathcal{R}$ and $\mathcal{B}^{\prime }$, we can always construct an infinite $\rightarrow _{\mathcal{R}/\mathcal{B}}$-reduction as in Example 4. Thus, $\mathcal{R}$ is not relatively terminating w.r.t. $\mathcal{B}$.

## Relative Termination and Minimality

A DP chain  is said to be *minimal* if every $t_i^\sharp$ is terminating w.r.t. $\mathcal{R}$. It is well known that absence of infinite minimal $(\mathsf {DP}(\mathcal{R}),\mathcal{R})$-chains implies absence of infinite $(\mathsf {DP}(\mathcal{R}),\mathcal{R})$-chains and thus termination of $\mathcal{R}$. A couple of techniques, namely *usable rules* and the *subterm criterion*, have been proposed to prove absence of infinite minimal chains [13].

Unfortunately, the minimality property (in terms of the standard DP framework) cannot be assumed on DP problems produced by our relative termination criteria. Therefore, usable rules and subterm criterion are not generally applicable.

### *Example 8*

Consider the following TRSs $\mathcal{R}$ and $\mathcal{B}$:

$$\begin{aligned} \mathcal{R}&= \{\, \mathsf {f}(\mathsf {s}(x)) \rightarrow \mathsf {f}(x)\, \}&\mathcal{B}&= \{\, \mathsf {inf} \rightarrow \mathsf {s}(\mathsf {inf})\, \} \end{aligned}$$

Theorem 2 yields the DP problem $(\{ \mathsf {f}^\sharp (\mathsf {s}(x)) \rightarrow \mathsf {f}^\sharp (x) \}, \mathcal{R}\cup \mathcal{B})$, which satisfies the subterm criterion in the argument of $\mathsf {f}^\sharp$. Moreover, since no rule is *usable* from the dependency pair $\mathsf {f}^\sharp (\mathsf {s}(x)) \rightarrow \mathsf {f}^\sharp (x)$, the usable rule technique would yield the DP problem $(\{ \mathsf {f}^\sharp (\mathsf {s}(x)) \rightarrow \mathsf {f}^\sharp (x) \}, \emptyset )$, which any standard technique proves finite. However, $\mathcal{R}/\mathcal{B}$ is not terminating as the following infinite reduction exists:

$$\begin{aligned} \mathsf {f}(\mathsf {s}(\mathsf {inf})) \rightarrow _\mathcal{R}\mathsf {f}(\mathsf {inf}) \rightarrow _\mathcal{B}\mathsf {f}(\mathsf {s}(\mathsf {inf})) \rightarrow _\mathcal{R}\mathsf {f}(\mathsf {inf}) \rightarrow _\mathcal{B}\cdots \end{aligned}$$

Nonetheless, we will show that both the subterm criterion and usable rules are still applicable when $\mathcal{B}$ satisfies the following condition:

### **Definition 8**

(*quasi-termination* [7]) We say that a TRS $\mathcal{R}$ is *quasi-terminating* iff the set  is finite for every term *s*.

First, we naturally extend the notion of minimality to relative termination.

### **Definition 9**

(*relative DP problem*) A *relative DP problem* is a triple of TRSs, written $(\mathcal{P},\mathcal{R}/\mathcal{B})$. A $(\mathcal{P},\mathcal{R}/\mathcal{B})$-*chain* is a possibly infinite sequenceand is called *minimal* if every $t_i^\sharp$ is $\mathcal{R}/\mathcal{B}$-terminating. The relative DP problem is *finite* if it admits no infinite minimal chain.

Clearly, finiteness of $(\mathsf {DP}(\mathcal{R}),\mathcal{R}/\mathcal{B})$ is equivalent to that of $(\mathsf {DP}(\mathcal{R}),\mathcal{R}\cup \mathcal{B})$. Hence our previous results hold as well for the corresponding relative DP problems.

### Relative Subterm Criterion

The subterm criterion is applicable when the base $\mathcal{B}$ is quasi-terminating. More precisely, we require that ${\vartriangleright }/{\rightarrow _\mathcal{B}}$ is terminating, i.e., there exist no infinite sequence of the following form:

3 $$\begin{aligned} s_1 \vartriangleright t_1 \rightarrow _\mathcal{B}^{*} s_2 \vartriangleright t_2 \rightarrow _\mathcal{B}^{*} \cdots \end{aligned}$$

#### **Lemma 7**

If $\mathcal{B}$ is quasi-terminating, then ${\vartriangleright }/{\rightarrow _\mathcal{B}}$ is terminating.

#### *Proof*

We prove the claim by contradiction. Assume that there is an infinite sequence of form (3). Since $\vartriangleright$ is well-founded, the sequence must also contain infinitely many $\rightarrow _\mathcal{B}$ reductions. We obtain an infinite sequence (note that $\vartriangleright$ is transitive):

$$\begin{aligned} u_1 \vartriangleright v_1 \rightarrow _\mathcal{B}^+ u_2 \vartriangleright v_2 \rightarrow _\mathcal{B}^+ \cdots \end{aligned}$$

By the definition of $\vartriangleright$, $u_i = C_i[v_i]$ for some nonempty context $C_i$. We obtain the following infinite $\rightarrow _\mathcal{B}$ sequence:

$$\begin{aligned} u_1 = C_1[v_1] \rightarrow _\mathcal{B}^+ C_1[u_2] = C_1[C_2[v_2]] \rightarrow _\mathcal{B}^+ \cdots \end{aligned}$$

which yields infinitely many different $\mathcal{B}$-reducts of $u_1$. This contradicts the assumption that $\mathcal{B}$ is quasi-terminating. $\square$

Now we introduce some notions which are needed to formally describe the subterm criterion.

#### **Definition 10**

(*simple projection*) A *simple projection* $\pi$ is a mapping that assigns each *n*-ary symbol $f^\sharp$ one of its argument position $i \in \{ 1, \dots , n \}$. For a term $t^\sharp = f^\sharp (t_1,\dots ,t_n)$, we denote by $\pi (t^\sharp )$ the term $t_i$ with $i = \pi (f^\sharp )$. For a relation $\sqsupset$ on terms, $\sqsupset ^\pi$ is defined as follows: $s \sqsupset ^\pi t$ iff $\pi (s) \sqsupset \pi (t)$.

#### **Theorem 3**

(relative subterm criterion) Let $(\mathcal{P},\mathcal{R}/\mathcal{B})$ be a relative DP problem such that ${\vartriangleright }/{\rightarrow _\mathcal{B}}$ is terminating, and let $\pi$ be a simple projection such that ${\mathcal{P}} \subseteq {\trianglerighteq ^\pi }$. Then, $(\mathcal{P},\mathcal{R}/\mathcal{B})$ is finite if $({\mathcal{P}} \setminus {\vartriangleright ^\pi }, \mathcal{R}/\mathcal{B})$ is finite.

#### *Proof*

We prove the claim by contradiction. Assume that there exists a minimal infinite chain of the form:such that $\pi (s_i^\sharp ) \vartriangleright \pi (t_i^\sharp )$ for infinitely many choices of *i*. Using the discussion in [13, Theorem11], we obtain the following infinite sequence:

4 $$\begin{aligned} \pi (t^\sharp ) \rightarrow _{\mathcal{R}\cup \mathcal{B}}^{*} \pi (s_{i_1}^\sharp ) \vartriangleright \pi (t_{i_1}^\sharp ) \rightarrow _{\mathcal{R}\cup \mathcal{B}}^{*} \pi (s_{i_2}^\sharp ) \vartriangleright \pi (t_{i_2}^\sharp ) \rightarrow _{\mathcal{R}\cup \mathcal{B}}^{*} \cdots \end{aligned}$$

Since every proper subterm of *t* is $\mathcal{R}/\mathcal{B}$-terminating, so is $\pi (t^\sharp )$. Moreover, $\vartriangleright$ preserves the termination property. Hence, the infinite sequence (4) cannot contain infinitely many $\rightarrow _\mathcal{R}$ steps, and thus we obtain an infinite ${\vartriangleright } \cdot {\rightarrow _\mathcal{B}^{*}}$ sequence. This contradicts the assumption that ${\vartriangleright }/{\rightarrow _\mathcal{B}}$ is terminating.$\square$

### Relative Usable Rules

Now we prove that the quasi-termination condition also enables the usable rules technique. We roughly follow the proof by Hirokawa and Middeldorp [15] for the non-relative case, but in the following definition we introduce a trick to handle $\mathcal{B}$-reductions, and use more convenient notations.

#### **Definition 11**

Let $\mathcal{R}$ be a finite TRS, $\mathcal{B}$ a quasi-terminating TRS, and $\mathcal{U}_\mathcal{F}$ a set of function symbols. For an $\mathcal{R}/\mathcal{B}$-terminating term *s*, we define $\overline{s}$ and $\widehat{s}$ as follows: $\overline{s} = \widehat{s} = s$ if *s* is a variable, and for $s = f(s_1,\dots ,s_n)$,

$$\begin{aligned} \overline{s}&= {\left\{ \begin{array}{ll} f(\overline{s_1},\dots ,\overline{s_n}) &{}\text {if } f \in \mathcal{U}_\mathcal{F}\\ \mathsf {list}(\{\,\widehat{t} \mid s \rightarrow _\mathcal{B}^{*} t\,\}) &{}\text {if } f \notin \mathcal{U}_\mathcal{F}\end{array}\right. }\\ \widehat{s}&= {\left\{ \begin{array}{ll} f(\overline{s_1},\dots ,\overline{s_n}) &{}\text {if } f \in \mathcal{U}_\mathcal{F}\\ \mathsf {list}( \{f(\overline{s_1},\dots ,\overline{s_n})\} \cup \{\,\overline{t} \mid s \rightarrow _\mathcal{R}t\,\}) &{}\text {if } f \notin \mathcal{U}_\mathcal{F}\end{array}\right. } \end{aligned}$$

Here, $\mathsf {list}(\{s_1,\dots ,s_n\})$ denotes the term $s_1 \circ \cdots \circ s_n$, where $\circ \notin \mathcal{F}$ is a fresh binary symbol and elements are sorted w.r.t. an arbitrary but fixed total order on terms.

It is easy to see that $\overline{s}$ and $\widehat{s}$ are well defined for every $\mathcal{R}/\mathcal{B}$-terminating *s*. Note also that $\overline{s}$ is a finite term due to the assumption that $\mathcal{B}$ is quasi-terminating. To ease readability, the rest of this section assumes $\mathcal{R}$ to be finite and $\mathcal{B}$ quasi-terminating, without explicitly stating it.

A key property of Definition 11 is that $\mathcal{R}$- and $\mathcal{B}$-reductions below a non $\mathcal{U}_\mathcal{F}$ symbol can be simulated by the following TRS, which is traditionally called ${\mathcal{C}_\mathcal{E}}$ (w.r.t. $\circ$).

$$\begin{aligned} {\mathcal{C}_\mathcal{E}}= \{\, x \circ y \rightarrow x,\, x \circ y \rightarrow y\,\} \end{aligned}$$

#### **Lemma 8**

If *s* is $\mathcal{R}/\mathcal{B}$-terminating, , and $\mathsf {root}(s) \notin \mathcal{U}_\mathcal{F}$, then .

#### *Proof*

First consider . In this case, we have . Hence, by properly applying ${\mathcal{C}_\mathcal{E}}$ rules we obtainNext consider . In this case, we have
$$\square $$

The following auxiliary result for substitutions is analogous to Hirokawa and Middeldorp [15], but the proof needs a little modification for the distinction of $\overline{s}$ and $\widehat{s}$. We say that a substitution $\sigma$ is $\mathcal{R}/\mathcal{B}$-*terminating* if $x\sigma$ is $\mathcal{R}/\mathcal{B}$- terminating for every variable *x*. For an $\mathcal{R}/\mathcal{B}$- terminating substitution $\sigma$, $\overline{\sigma }$ denotes the substitution that maps each *x* to $\overline{x\sigma }$.

#### **Lemma 9**

If $\sigma$ and $s\sigma$ are $\mathcal{R}/\mathcal{B}$-terminating, then . If moreover $s \in \mathcal{T}(\mathcal{U}_\mathcal{F},\mathcal{V})$, then $\overline{s\sigma } = s\overline{\sigma }$.

#### *Proof*

We prove the claim by induction on the structure of *s*. If $s \in \mathcal{V}$ then trivially $\overline{s\sigma } = s\overline{\sigma }$. Assume that $s = f(s_1,\dots ,s_n)$. We have

$$\begin{aligned} \overline{s\sigma } = \overline{f(s_1,\dots ,s_n)\sigma } = \overline{f(s_1\sigma , \dots , s_n\sigma )} \end{aligned}$$

Now, we consider the following two cases:

1. If $f \in \mathcal{U}_\mathcal{F}$, then by definition $\overline{s\sigma } = f(\overline{s_1\sigma },\dots ,\overline{s_n\sigma })$.
2. If $f \notin \mathcal{U}_\mathcal{F}$, then we have

Using the induction hypotheses, we obtainThus we conclude . If $s \in \mathcal{T}(\mathcal{U}_\mathcal{F},\mathcal{V})$, then case 2 does not occur and we obtain $\overline{s\sigma } = s\overline{\sigma }$. $\square$

Now we define the notion of usable rules.

#### **Definition 12**

Let $\mathcal{U}\subseteq \mathcal{R}\cup \mathcal{B}$ and $\mathcal{U}_\mathcal{F}\subseteq \mathcal{F}$. Elements in $\mathcal{U}$ ($\mathcal{U}_\mathcal{F}$) are called *usable rules* (*usable symbols*) in a relative DP problem $(\mathcal{P},\mathcal{R}/\mathcal{B})$, if the following conditions hold:

1. If $l \rightarrow r \in \mathcal{R}\cup \mathcal{B}$ and $\mathsf {root}(l) \in \mathcal{U}_\mathcal{F}$, then $l \rightarrow r \in \mathcal{U}$.
2. If $$l \rightarrow r \in \mathcal{U}\cup \mathcal{P}$$, then $$r \in \mathcal{T}(\mathcal{U}_\mathcal{F},\mathcal{V})$$.

Let us say that a position *p* in a term *s* is *usable* if $\mathsf {root}(s|_q) \in \mathcal{U}_\mathcal{F}$ for every proper prefix *q* of *p*. The following lemma is straightforward, which is also a corollary of Hirokawa and Middeldorp [15, Lemma 18].

#### **Lemma 10**

If *p* is a usable position in *s* and $s[t]_p$ is $\mathcal{R}/\mathcal{B}$-terminating, then $\overline{s[t]_p} = \overline{s}[\,\overline{t}\,]_p$.

#### **Lemma 11**

Let $\mathcal{U}$ and $\mathcal{U}_\mathcal{F}$ satisfy the conditions of Definition 12. If *s* is $\mathcal{R}/\mathcal{B}$-terminating and , then .

#### *Proof*

Let  and $t = s[r\sigma ]_p$ for some $l \rightarrow r \in \mathcal{R}\cup \mathcal{B}$. We distinguish the following two cases:

- Suppose that *p* has a usable prefix *q* such that $\mathsf {root}(s|_q) \notin \mathcal{U}_\mathcal{F}$. We have , and Lemma 8 entails . Using Lemma 10, we get
- Otherwise, *p* is a usable position and $\mathsf {root}(l) \in \mathcal{U}_\mathcal{F}$. By condition 1 we have $l \rightarrow r \in \mathcal{U}$. By Lemma 9, we have . Further, we know $r \in \mathcal{T}(\mathcal{U}_\mathcal{F},\mathcal{V})$ by condition 2 of Definition 12. Hence, by Lemma 9, we have $r\overline{\sigma } = \overline{r\sigma }$. Finally, using Lemma 10, we obtain 
$$\square $$

Finally, we present the main result of this section. Using the preceding lemmas, the main theorem is proven analogously to the standard case.

#### **Theorem 4**

(relative usable rules) Let $(\mathcal{P},\mathcal{R}/\mathcal{B})$ be a relative DP problem such that $\mathcal{R}$ is finite and $\mathcal{B}$ is quasi-terminating. Let $\mathcal{U}$ satisfy the conditions of Definition 12, and $(\succsim ,\succ )$ be a reduction pair such that $\mathcal{P}\cup \mathcal{U}\cup {\mathcal{C}_\mathcal{E}}\subseteq {\succsim }$. Then, $(\mathcal{P},\mathcal{R}/\mathcal{B})$ is finite if and only if the relative DP problem $(\mathcal{P}\setminus {\succ },\mathcal{R}/\mathcal{B})$ is finite.

#### *Proof*

The “only-if” direction is trivial. For the “if” direction, consider a minimal infinite $(\mathcal{P},\mathcal{R}/\mathcal{B})$-chain:5where $l_i^\sharp \rightarrow r_i^\sharp \in \mathcal{P}$ for every $i = 0,1,2,\cdots$. The minimality assumption entails that $r_{i}^\sharp \sigma _{i}$ is $\mathcal{R}/\mathcal{B}$-terminating, and thus $\overline{r_{i}^\sharp \sigma _{i}}$ is defined. Using Lemma 11, we have . Since $\mathcal{U}\cup {\mathcal{C}_\mathcal{E}}\subseteq {\succsim }$ and $\succsim$ is closed under contexts and substitutions, we obtainFor $i = 1,2,\dots$, we have  by Lemma 9. By condition 2 of Definition 12 we know $r_i^\sharp \in \mathcal{T}(\mathcal{U}_\mathcal{F},\mathcal{V})$ and hence $r_i^\sharp \overline{\sigma _i} = \overline{r_i^\sharp \sigma _i}$ by Lemma 9. We obtainSince $(\succsim ,\succ )$ is a reduction pair, we cannot have $l_i^\sharp \succ r_i^\sharp$ for infinitely many *i*. Thus, after some point (5) contains no  steps, which is a desired minimal infinite $(\mathcal{P}\setminus {\succ },\mathcal{R}/\mathcal{B})$-chain. $\square$

It is well known that, unfortunately, the quasi-termination condition is undecidable [7]. In our implementation, we only use a trivial sufficient condition, *non- size increasingness*. We admit that this is quite restrictive, and thus leave it for future work to find a more useful syntactic condition for this purpose.

From Example 8, it is clear that the usable rule argument does not apply to the rules in $\mathcal{B}$ if they are not quasi-terminating. Nonetheless, we conjecture that the usable rule argument may be still applicable to the rules in $\mathcal{R}$.

## Experimental Evaluation

We implemented our technique into the termination prover NaTT (since ver. 1.2). The implementation works as follows: It first checks if the rules in $\mathcal{B}$ satisfy the two conditions of Theorem 2. If there is a rule that violates the conditions, then it moves the rule into $\mathcal{B}^{\prime }$, and repeats the procedure until a partition satisfying the conditions of Corollary 3 is obtained. Then it enables the usable rules technique^6^ if the remaining $\mathcal{B}$ rules are non-size increasing, although it is not often the case for the present benchmarks.

Afterwards, we obtain a standard dependency pair problem, on which existing reduction pair processors can be applied. In the experiments, we use the following reduction pairs for Proposition 2:

- *polynomial interpretations* [2, 26], extended for a limited form of negative coefficients [14],
- the *lexicographic path order* [20] with *argument filtering* [2],
- the *weighted path order* with partial status [35], and
- (2- or 3-dimensional) *matrix interpretations* [8].

As monotone reduction pairs for Proposition 3, we use polynomial and matrix interpretations with top-left elements of coefficients being at least 1 [8].

In the following, we show the significance of our technique through an experimental evaluation. The experiments were run on a server equipped with a quad-core Intel Xeon E5-3407v2 processor running at a clock rate of 2.40 GHz and 32 GB of main memory. NaTT uses z3  4.3.2 7 as a back-end SMT solver.

**Table 1:** Experiments

Method	TPDB relative (44)				AG01 $+$ relative (44)			
	${\text {Yes}}$	Maybe	T.O.	${\text {Time}}$	${\text {Yes}}$	${\text {Maybe}}$	${\text {T.O.}}$	${\text {Time}}$
Theorem 2	4	40	0	0.81	29	15	0	5.13
Corollary 3	6	28	0	37.87	29	15	0	5.05
Proposition 3	23	17	4	406.05	9	35	0	6.62
Prop. 3 + Cor. 3	25	11	8	505.52	35	9	0	13.03
AProVE	27	(no: 8)	9	756.66	14	0	30	1959.91

The first test set consists of the 44 examples in the “TRS Relative” category of the *termination problem database* (TPDB) 9.0.^8^ The results are presented in the left half of Table 1. The “yes” column indicates the number of successful termination proofs, “maybe” indicates the number of failures, “T.O.” indicates the number of timeouts, which is set to 60 s, and “time” indicates the total run time. In the first two rows, we directly apply Theorem 2 and Corollary 3, and then apply the aforementioned reduction pairs. We observe that they are of limited applicability on this set of problems due to the non-duplication and dominance conditions. Nonetheless, Corollary 3 could prove the relative termination of two problems^9^ that no tools participating in the *Termination Competition 2015* were able to prove. For comparison, we include results for rule removal processors by matrix interpretations in the third row.

We also prepared 44 examples by extending benchmarks by Arts and Giesl [3] with the random number generator $\mathcal{B}_\mathsf {rand}$ or the commutative list specification $\mathcal{B}_\mathsf {comlist}$, which is added to TPDB 10.0. The results are presented in the right half of Table 1. In these examples, the power of our method should be clear. Theorem 2 is already able to prove the relative termination of 29 examples, while AProVE succeeds only in 14 examples.

The DP framework allows the combination of termination proving techniques. In the fourth row, we combine the rule removal processors and the technique presented in this paper. This combination indeed boosts the power of NaTT; e.g., by combining Proposition 3 and Corollary 3, NaTT can prove relative termination for a total of 60 examples (out of 88), while AProVE can only prove it for 41 examples.^10^ Therefore, we conclude that our technique improves the state-of-the-art methods for proving relative termination.

Although NaTT won the “TRS Relative” category in the Termination Competition 2015, it is clear that the mostly artificially crafted 88 examples in the category are limited as a fair benchmark set (especially compared to the “TRS Standard” category containing almost 1500 examples). We hope that new relative termination problems stemming from different applications will alleviate this problem in the near future.

## Related Work

One of the most comprehensive works on relative termination is Geser’s Ph.D. thesis [9]. Indeed, one of his main results is formulated in Proposition 3 of Sect. 2. A similar technique has been used, e.g., to prove confluence in [16]. Of course dependency pairs are not considered in [9] since they were introduced almost a decade later. Dependency pairs are considered in [8], but they are mainly used to prove termination of a TRS $\mathcal{R}$ by proving termination of $\mathsf {DP}(\mathcal{R})/\mathcal{R}$, which is quite a different purpose from ours.

Giesl and Kapur [10] adapted the dependency pair method for proving termination of *equational rewriting*, a special case of relative termination where the base is symmetric ($\mathcal{B}= \mathcal{B}^{-1}$). For more specific *associative-commutative (AC)* rewriting, a number of papers exist ([1, 25, 27], etc.). The key technique behind them is to compute an extension of $\mathcal{R}$ w.r.t. the considered equations. This operation allows symbols in $\mathcal{B}$ (e.g., AC symbols) to be defined also in $\mathcal{R}$, and hence no counterpart of the dominance condition is required. However, such extensions are computable only for certain equations (e.g., AC), and thus they are not appropriate in our setting, where an arbitrary base $\mathcal{B}$ is considered.

The closer approach is Iborra et al. [18], where the main aim was proving termination of narrowing [17, 31] by proving relative termination of a corresponding rewrite relation, similarly to [29, 33]. In [18], a first attempt to reduce relative termination to a DP problem is made by requiring $\mathcal{R}$ and $\mathcal{B}$ to form a so-called *hierarchical combination (HC)* [30], i.e., $\mathcal{D}_\mathcal{R}\cap \mathcal{F}_\mathcal{B}= \emptyset$. Unfortunately, we found that Iborra et al. [18, Theorem 5] was incorrect since requiring $\mathcal{B}$ to be non-duplicating is also necessary. In fact, Example 4 is a counterexample to Iborra et al. [18, Theorem 5]. The present paper corrects and significantly extends [18]; namely, all results in Sects. 3, 5, and 6 are new, and those in Sect. 4 correct and extend the previous result of Iborra et al. [18]. Note also that the HC condition of Iborra et al. [18] is a special case of our dominance condition. Moreover, we developed an implementation that allowed us to experimentally verify that our technique indeed pays off in practice.

This paper is a revised and extended version of [19]. Besides adding more explanations and missing proofs (most notably in Sect. 6.2), we found and filled a gap in the original proofs.^11^ To elegantly correct it, we now present Sect. 3 in a more essential way, and reformulate the original claims in Sects. 4 and 5 accordingly. Moreover, we improved the claim of the main result (Theorem 2) in order to show soundness and completeness (before only soundness was considered).

## Conclusion

In this paper, we have introduced a new approach to proving relative termination by reducing it to DP problems. The relevance of such a result should be clear, since it allows one to prove relative termination by reusing many existing techniques and tools for proving termination within the DP framework. Indeed, such an approach was included in the RTA List of Open Problems (Problem #106). To the best of our knowledge, this work makes the first significant contribution to positively answering this problem. Moreover, as shown in Sect. 7, our method is competitive w.r.t. state-of-the-art provers for the problems in the TPDB, and is clearly superior for examples including the generation of random values or the simulation of extra-variables, as discussed in Sect. 1.

As future work, we plan to improve the precision of our technique by extending the DP framework to be more suitable for proving relative termination. We will also continue the research on finding less restrictive conditions on $\mathcal{R}$ and $\mathcal{B}$ so that the technique becomes more widely applicable.

## Footnotes

1 Available from URL http://www.termination-portal.org/.

2
http://www.win.tue.nl/rtaloop/.

3 Available at http://www.trs.cm.is.nagoya-u.ac.jp/NaTT/.

4 Note that our notion of “proof ordering” is not related to that in [5, 6].

5 Actually, $\succsim$ can be an arbitrary reflexive relation.

6 Unfortunately, the subterm criterion is not implemented in NaTT.

7 Available at https://github.com/Z3Prover/z3/.

8 Available at http://termination-portal.org/wiki/TPDB.

9 For one of the two problems, the union is terminating.

10 For four examples, AProVE proved relative termination but NaTT failed. In this cases, AProVE used *semantic labeling* [36], which is currently not implemented in NaTT.

11 In [19, Definition 3], $\succ _{\mathcal{R}/\mathcal{B}}$ should be defined as $(\rightarrow _{\mathcal{R}/\mathcal{B}}\cdot \trianglerighteq )^+$ rather than $(\rightarrow _{\mathcal{R}/\mathcal{B}}\cup \vartriangleright )^+$. The latter definition is not well-founded if ${\rightarrow _\mathcal{B}^+} \cap {\vartriangleleft } \ne \emptyset$. A straightforward correction would require a tedious workaround to prove [19, Lemma 5].
